# Near-infrared (NIR) perfusion angiography in minimally invasive colorectal surgery

**DOI:** 10.1007/s00464-014-3432-y

**Published:** 2014-02-25

**Authors:** Frederic Ris, Roel Hompes, Chris Cunningham, Ian Lindsey, Richard Guy, Oliver Jones, Bruce George, Ronan A. Cahill, Neil J. Mortensen

**Affiliations:** 1 Department of Colorectal Surgery, Oxford University Hospitals, Churchill Hospital, Old Road, Oxford, OX3 7LJ Headington UK; 2 Service of Visceral Surgery, Geneva University Hospital and Medical School, 4 rue Gabrielle-Perret-Gentil, Geneva, 1211 Switzerland; 3 Department of Colorectal Surgery, Beaumont Hospital, Beaumont Road, Dublin, Ireland

**Keywords:** Laparoscopy, Colorectal resection, Anastomotic leak, Near infrared (NIR) laparoscopy, Indocyanine green (ICG), Perfusion

## Abstract

**Background:**

Anastomotic leakage is a devastating complication of colorectal surgery. However, there is no technology indicative of in situ perfusion of a laparoscopic colorectal anastomosis.

**Methods:**

We detail the use of near-infrared (NIR) laparoscopy (PinPoint System, NOVADAQ, Canada) in association with fluorophore [indocyanine green (ICG), 2.5 mg/ml] injection in 30 consecutive patients who underwent elective minimally invasive colorectal resection using the simultaneous appearance of the cecum or distal ileum as positive control.

**Results:**

The median (range) age of the patients was 64 (40–81) years with a median (range) BMI of 26.7 (20–35.5) kg/m^2^. Twenty-four patients had left-sided resections (including six low anterior resections) and six had right-sided resections. Of the total, 25 operations were cancer resections and five were for benign disease [either diverticular strictures (*n* = 3) or Crohn’s disease (*n* = 2)]. A high-quality intraoperative ICG angiogram was achieved in 29/30 patients. After ICG injection, median (range) time to perfusion fluorescence was 35 (15–45) s. Median (range) added time for the technique was 5 (3–9) min. Anastomotic perfusion was documented as satisfactory in every successful case and encouraged avoidance of defunctioning stomas in three patients with low anastomoses. There were no postoperative anastomotic leaks.

**Conclusion:**

Perfusion angiography of colorectal anastomosis at the time of their laparoscopic construction is feasible and readily achievable with minimal added intraoperative time. Further work is required to determine optimum sensitivity and threshold levels for assessment of perfusion sufficiency, in particular with regard to anastomotic viability.

**Electronic supplementary material:**

The online version of this article (doi:10.1007/s00464-014-3432-y) contains supplementary material, which is available to authorized users.

Colorectal resection is a common operation with more than 600,000 procedures performed each year in the US alone. Anastomotic leakage remains its most concerning complication, with often devastating clinical results for the patient and considerable economic consequence for the healthcare provider [1]. While relatively uncommon in any single surgeon’s practice, average leak rates of 1–3 % for ileocolic anastomoses and up to 10–20 % for low colorectal anastomoses impact adversely on postoperative outcomes worldwide [2, 3]. These incidences have persisted despite widespread and increasing uptake of laparoscopic approaches that have made a significant and positive impact on postoperative complication rates [4].

Various patient- and procedure-related variables have been implicated as risk factors for anastomotic breakdown, although absolute prediction of its occurrence in any one individual remains difficult [5]. Although a common determining factor of viability is adequate arterial perfusion to ensure sufficient local tissue oxygenation [6], there is currently no accepted method to assess the viability of a colorectal anastomosis in situ after its laparoscopic construction. Common practice nowadays is simply to use crude visual assessment of the transected proximal bowel at the time of specimen extraction and thereafter inspection of the whole anastomosis after stapling. A more sophisticated capability to view the actual vascular and microvascular perfusion at the time of reanastomosis may increase confidence in the technical perfection or, alternatively, indicate perfusion deficiency and so prompt reconstruction or even abandonment of anastomosis. Here, we report the use of near-infrared (NIR) laparoscopic technology to confirm and document viability and perfusion of digestive anastomoses by means of an on-table, real-time fluorophoric [indocyanine green (ICG)] angiogram in a series of consecutive patients who underwent laparoscopic colorectal resection with primary anastomosis. This builds on the earlier recently published experience in which similar technology was employed endoluminally [7, 8] and within the Da Vinci robot platform [9] and indeed for nongastrointestinal indications [10, 11].

## Methods

### Patient selection and ethical approval

Full ethical approval for this study was granted after application to an Independent Research Ethics Committee (North London REC-2, ethical committee approval No. 10/H0724/13), and institutional, departmental, and external peer approval of the entire protocol was obtained. Thirty consecutive patients who were to undergo elective colonic or rectal resection for either colorectal cancer or benign pathology consented in full before their agreed recruitment; with all being informed that inclusion in the study was voluntary and simply additive to routine care.

### ICG

ICG is supplied as sterile water-soluble lyophilized powder (25 mg, ICG Pulsion®, Pulsion Medical Systems, Munich, Germany) with the formula C_43_H_47_N_2_O_6_S_2_Na [12]. It is an anhydro-3,3,3′,3′-tetramethyl-1,1′-di-(4-sulfobutyl)-4,5,4′,5′-dibenzoindotricarbocyanine hydroxide sodium salt with molecular weight of 775 Da. A fluorophore in response to NIR irradiation, it absorbs light between 790 and 805 nm and re-emits it with an excitation wavelength of 835 nm [13]. ICG half-life is 3–5 min and it is eliminated by the liver in 15–20 min. These properties in conjunction with the absence of any native biological fluorescence within these wavelengths make ICG an ideal agent for the acquisition of high-quality images of both the circulatory and lymphatic systems [14]. While generally very safe for intravenous use, vasovagal or allergic reactions can occur and the incidence of fatality due to its use is estimated as one per 333,000.

### Laparoscopic NIR fluorescence imaging system

A prototype NIR laparoscopic system (PinPoint Endoscopic Fluorescence Imaging System, NOVADAQ, Mississauga, ON, Canada) was used to provide high-definition white light, NIR irradiation, and back-filtration specifically tuned for ICG. This system also provides a superimposition of both modalities using false-coloring technology allowing an enhanced real-time appreciation of dynamic perfusion without loss of the standard white light view. Though not yet CE-marked, the system was used with approval after specific MHRA and ethics clearance. ICG injection was performed immediately after anastomosis construction, with a segment of unoperated right colon or ileum used as positive comparator in each case.

### Operative procedure

Colorectal resection was performed in a standardized fashion by multiport laparoscopy for every case, utilizing a fully laparoscopic technique for left-sided resections (classified as either high or low anterior resection depending on whether the double-stapled colorectal anastomosis was above or below the peritoneal reflection) and a laparoscopy-assisted approach (stapled or handsewn extracorporeal anastomosis with subsequent relaparoscopy) for right-sided resections. A medial to lateral dissection technique was used and the splenic flexure was mobilized routinely for left-sided resections.

### Data collection

Patient characteristics, intraoperative parameters, and postoperative outcomes were collected prospectively. Perfusion images were recorded and qualitatively judged in real-time. Specific NIR criteria related to the timing and quantity of injection as well as anastomotic fluorescence intensity and persistence were measured and any change in operative protocol prompted by the ICG angiogram was noted. Intraoperative adverse reactions were recorded in real-time and postoperative complications were classified according to the Dindo–Clavien classification [15].

### Statistical analysis

**Fig. 1:**
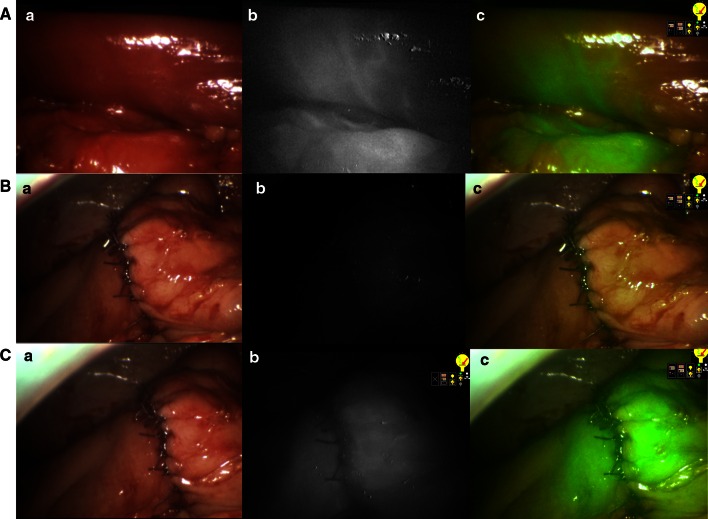
NIR perfusion assessment in laparoscopic right hemicolectomy. **a** Normal light. **b** Near-infrared fluorescence. **c** Superposition of NIR and normal light in green. **A** Intraoperative photos showing a clear demarcation line after vessel division. **B** Ileotransverse anastomosis before IDC injection, showing no fluorescence. **C** Perfusion assessment of the ileotransverse anastomosis

**Fig. 2:**
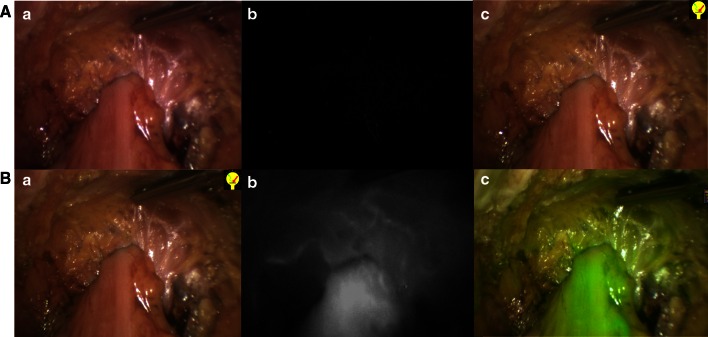
NIR perfusion assessment in laparoscopic low anterior resection. **a** Normal light. **b** Near-infrared fluorescence. **c** Superposition of NIR and normal light in green. **A** Colorectal end-to-end anastomosis before IDC injection, showing no fluorescence. **B** Perfusion assessment of the colorectal anastomosis

**Fig. 3:**
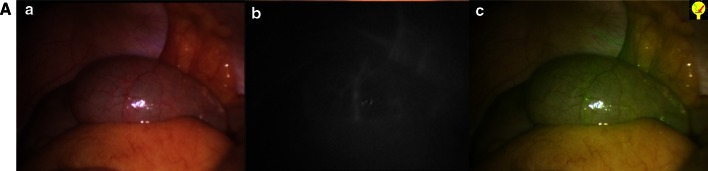
Positive control. **a** Normal light. **b** Near-infrared fluorescence. **c** Superposition of NIR and normal light in green. **A** Image of the normal cecum after IDC injection

Results were expressed as median (range). Continuous variables were compared with the Student *t* test and categorical variables with the χ*χ* ^2^ test. All tests were conducted using the standard α level of 0.05 to indicate statistical significance (Figs. 1, 2, 3).

## Results

Thirty consecutive patients were recruited for the study over a 5-month period. Patient and disease characteristics are given in Table 1 and operation parameters are given in Table 2. An intraoperative ICG perfusion angiogram of the anastomosis and supporting colorectal mesentery was achieved in every patient except one (patient no. 2) when the system failed to detect any visible fluorescence. The time of visible ICG fluorescence at the anastomosis was less than 1 min in each successful case, the median time being 35 s (see Table 3 and video 1). Fluorescence persisted at the site of interest for more than 3 min, with a residual weak signal visible up to 30 min after the initial injection (video 2). Once it starts to fade, a second ICG injection at least 5 min after the first injection could refluoresce the target tissue. With the normal, nearly adjacent cecum or terminal ileum, in case of right hemicolectomy, used as a comparative positive control, the perfusion appearances of the colorectal anastomosis were judged satisfactory in every patient and did not prompt revision or other technical adjustment of any anastomosis. A defunctioning stoma (normally our preference for anastomoses within 8 cm of the anal verge), was avoided in three of six patients in part because of the confidence imparted by the perfusion angiogram. There was no need to redo the anastomosis in any patient based on the angiogram findings. The median added procedure time from using this technological modality was 4.5 (3–9) min, with the shorter times occurring with experience. There was no long-term or short-term morbidity related to ICG injection. None of the patients developed a problem related to the anastomosis (no leaks in this series), and the only postoperative morbidity (Table 4) was related to carbonarcosis due to analgesic drugs.

**Table 1:** Patient characteristics

*N*	30
Gender (M/F)	19/11
Age (years)	64 (40–79)
BMI (kg/m^2^)	26.7 (20-35.6)
Anesthetic risk	
ASA I	3 (10 %)
ASA II	25 (83 %)
ASA III	2 (7 %)
ASA IV	0 (0 %)
Indication for surgery	
Colorectal cancer	25 (83 %)
Diverticular disease	3 (10 %)
Crohn’s disease	2 (7 %)

Data are expressed as median (range) or number (%)

*BMI* body mass index, *ASA* American Society of Anesthesiologists

**Table 2:** Perioperative data

Laparoscopic high anterior resection	18 (60 %)
Laparoscopic low anterior resection	6 (20 %)
Laparoscopic right hemicolectomy	6 (20 %)
Conversion	3 (10 %)
Early	2 (7 %)
Late	1 (3.5 %)
Splenic flexure mobilization (high and low anterior resection, *n* = 25)	24 (96 %)
Protective ileostomy (low anterior resection)	3/6 (50 %)
Median length of procedure (min)	
Right hemicolectomy	146 (146–147)
High anterior resection	195 (95–296)
Low anterior resection	250 (188–270)

Data are expressed as median (range) or number (%)

**Table 3:** Perfusion assessment

Perioperative data	
Median length of the procedure (min)	4.5 (3–9)
Median time to reach the anastomosis (s)	35 (15–45)
Quality of the perfusion	
Good	29 (96 %)
Average	0
Bad	0
Technical failure	1 (4 %)
Change in anastomosis	0
Change in strategy (no diverting stoma in low anterior resection)	3 (50 %)

Data are expressed as median (range) or number (%)

**Table 4:** Postoperative data, hospital stay, and short- and long-term complications according to Clavien–Dindo classification

Hospital stay (days)	5 (2–8)
No complication	24
Complications (Clavien–Dindo)^a^	7
Grade I	3 (10 %)
Grade II	3 (10 %)
Grade IIIa	1 (3.5 %)
Grade IIIb	0
Grade IV	0
Reoperation	0
Anastomotic related complication	0
Long-term complication	0

Data are expressed as median (range) or number (%)

^a^There were seven complications in six patients

## Discussion

Although improved minimal access techniques and optimized perioperative care protocols [16] have greatly impacted short and intermediate postoperative outcomes, anastomotic complications (especially early postoperative anastomotic leaks) remain unpredictable and are often devastating. While a proximal defunctioning stoma may mitigate the consequences of anastomotic dehiscence, diversion also can negatively impact the patient in terms of psychological and physical functioning and with regard to actual complications in their formation and closure [17]. Furthermore, aside from acute dehiscence or breakdown, impaired perfusion can also contribute to intermediate or late stricture formation. Any means of minimizing or even avoiding anastomotic complications would justify considerable investment in time, effort, and direct investment. Current means of assessing anastomosis viability, however, relate only to simple inspection and checking mechanical integrity (i.e., donut assessment and air leak testing). At present, assessment of the collateral flow is possible only on the extracted proximal bowel and, of course, may not represent actual flow when the anastomosis is constructed within the abdominopelvic cavity. The perfusion angiogram clearly shows perfusion along the antimesocolic border of the colon proximal to the anastomosis for left-sided operations and distal to the anastomosis for right-sided resections. This is demonstrative of direct vascularization from the marginal artery, which lies along the mesocolic aspect of the colon and flow on this aspect is likely as sufficient. Equally, a mechanically intact anastomosis still may breakdown some days after its formation likely because of, at least in a proportion, vascular insufficiency.

Real-time NIR fluorescence angiography using ICG during laparoscopic colorectal surgery was proved feasible and reproducible with a minimum of added complexity. While a large prospective study is required to investigate its use in finding postoperative anastomotic leaks, the images appear compelling and seem at least to document technical sufficiency with regard to in situ vascularization of the proximal conduit at the time of anastomotic construction. Whether additional useful information could be gleaned by intraoperative assessment of the rectal stump prior to anastomosis or indeed the intraluminal aspect of the anastomosis after construction by transanal use via a sigmoidoscopy both during the operation and perhaps even on a daily basis after (in order to detect the failing anastomosis allowing corrective intervention prior to overt clinical deterioration) also requires further focused study. Finally, analytic measures to objectively quantify signal intensity are already evolving and require investment for their development and correlation with clinically important outcomes [18].

## Electronic supplementary material

Below is the link to the electronic supplementary material.

Supplementary material 1 (MOV 74093 kb)

Supplementary material 2 (MOV 73271 kb)
